# Optimal timing of adjuvant immunotherapy after radical cystectomy in muscle-invasive bladder cancer: from calendar-based to MRD-guided decision making

**DOI:** 10.3389/fonc.2026.1933232

**Published:** 2026-10-21

**Authors:** Nabih Naim, Ahmad Saad, Nesrine Mohamad, Joseph Kattan

**Affiliations:** Hematology Oncology Department, Saint Joseph University of Beirut, Beirut, Lebanon

**Keywords:** adjuvant immunotherapy, CtDNA, durvalumab, immune checkpoint inhibitor, minimal residual disease, muscle-invasive bladder cancer, nivolumab, pembrolizumab

## Abstract

The approval of adjuvant nivolumab and pembrolizumab following radical cystectomy and more recently perioperative durvalumab-based strategies represents a significant therapeutic advance in muscle-invasive bladder cancer (MIBC). However, a question that has received limited systematic attention concerns the optimal timing of immunotherapy initiation after surgery. Current recommendations are derived entirely from the eligibility windows of pivotal clinical trials, most notably CheckMate 274, AMBASSADOR and NIAGARA, which mandated randomization within approximately 120 days of cystectomy. These operational thresholds were designed to ensure postoperative recovery and were not prospectively validated as biologically optimal. This review critically examines the biological rationale underpinning timing considerations, appraises what clinical trials have actually studied, draws cautious parallels with adjuvant chemotherapy literature, and discusses how circulating tumour DNA (ctDNA) and minimal residual disease (MRD) detection may eventually redefine timing decisions. We conclude that no validated therapeutic window currently exists, that available evidence supports initiation within the studied timeframe when clinically feasible, and that prospective timing analyzes constitute a major unmet need in MIBC research.

## Introduction

Muscle-invasive bladder cancer (MIBC) carries a substantial risk of disease recurrence following radical cystectomy (RC), with approximately 50% of patients ultimately developing metastatic disease. The five-year overall survival (OS) for patients with pT3/T4 or node-positive disease remains below 40%, reflecting the high burden of occult micrometastatic dissemination at the time of surgery (1). The introduction of platinum-based neoadjuvant chemotherapy modestly improved survival outcomes, with a 5-8% absolute benefit in OS demonstrated in the landmark SWOG 8710 trial, yet real-world compliance with neoadjuvant regimens remains suboptimal, leaving the majority of patients to undergo surgery without systemic therapy (2).

Against this background, the emergence of immune checkpoint inhibitors (ICIs) as adjuvant therapy has fundamentally altered the post-cystectomy treatment landscape (3, 4). The phase III CheckMate 274 trial demonstrated that adjuvant nivolumab significantly prolonged disease-free survival (DFS) compared with placebo in patients with high-risk MIBC or upper tract urothelial carcinoma (UTUC) following RC or nephroureterectomy, with a hazard ratio (HR) of 0.70 overall (98.22% CI 0.55–0.90; p<0.001) and 0.55 in the PD-L1-positive subgroup (98.72% CI 0.35–0.85; p<0.001) (4). Subsequently, the AMBASSADOR trial (Alliance A031501) confirmed a significant DFS benefit with adjuvant pembrolizumab (HR 0.69; 95% CI 0.54–0.89; p=0.007) (3). These two studies included high-risk patients with or without neo-adjuvant chemotherapy. These results have led to regulatory approvals by the U.S. Food and Drug Administration and incorporation into major clinical practice guidelines (5, 6).

More recently, the therapeutic landscape has expanded beyond purely adjuvant approaches toward perioperative immunotherapy strategies. The phase III NIAGARA trial demonstrated improved outcomes with perioperative durvalumab combined with neoadjuvant cisplatin-based chemotherapy in cisplatin-eligible patients, while the VOLGA trial established the efficacy of perioperative durvalumab-based combinations in cisplatin-ineligible or cisplatin-declining populations. Similarly, perioperative pembrolizumab combined with enfortumab vedotin demonstrated significant clinical benefit in the KEYNOTE-905/EV-303 trial among cisplatin-ineligible patients and in the KEYNOTE-B15/EV-304 trial among cisplatin-eligible patients. Collectively, these studies have broadened the role of immunotherapy across the peri-cystectomy continuum (7–10).

Despite this progress, a critically underexplored question persists: when after RC should adjuvant ICI therapy be initiated? Although guidelines reflect the eligibility windows of pivotal trials, generally mandating treatment commencement within approximately 120 days of surgery, this temporal threshold was operationally defined rather than derived from prospective biological analyzes (5, 6, 11). In clinical practice, delayed recovery from major surgery, postoperative complications, ileus, wound dehiscence, and performance status concerns frequently cause delays beyond the initially anticipated timeframe (12, 13). Whether such delays meaningfully compromise therapeutic benefit remains unknown.

This review article critically evaluates the existing evidence concerning the timing of adjuvant immunotherapy after RC, examines the biological plausibility of timing-dependent effects, reviews what clinical trials have actually studied, and discusses the potential for biomarker-guided approaches to refine future practice (11). Throughout, we distinguish carefully between established evidence, emerging hypothesis, and expert opinion.

## Biological rationale for the timing of adjuvant immunotherapy

### A-Minimal residual disease and the adjuvant rationale

The fundamental rationale for adjuvant ICI therapy rests on the presumed presence of minimal residual disease (MRD), microscopic occult tumor deposits remaining after macroscopically complete surgery that cannot be detected by conventional imaging. In this context, immune checkpoint blockade is hypothesized to augment anti-tumor T-cell responses against residual clonogenic cells before clinically detectable relapse develops (14). The theoretical advantage of early initiation is that tumor burden is at its nadir and antigen presentation most tractable, facilitating immune-mediated clearance (14, 15). However, as discussed below, the post-cystectomy biological milieu may counteract these benefits if treatment is initiated during the period of maximal postoperative immune dysfunction (16, 17).

### B-Postoperative inflammatory response and immune dysregulation

Radical cystectomy induces a profound and reproducible systemic inflammatory response characterized by elevations in pro-inflammatory cytokines, including interleukin-6 (IL-6), tumor necrosis factor-alpha (TNF-α), and C-reactive protein, typically peaking within 24–72 hours postoperatively and persisting for up to two weeks. These cytokine changes reflect tissue injury and are amplified by the extent of the surgical dissection, lymphadenectomy, and urinary diversion (12, 13).

Simultaneously, major abdominal surgery is associated with a state of relative immunosuppression in the early postoperative period, characterized by impaired natural killer (NK) cell cytotoxic activity, reduced T-cell proliferative responses and lymphopenia, and expansion of myeloid-derived suppressor cells (MDSCs) changes that collectively suppress anti-tumor immune surveillance. Anesthetic agents and perioperative opioid administration independently contribute to this postoperative immunosuppression (17). These surgery-induced immune perturbations may theoretically impair the efficacy of ICI therapy if treatment is initiated during the period of maximal immune dysfunction, generally the first 4–6 weeks after major abdominal surgery (16). Conversely, excessive delay may allow residual tumor cells to re-establish immune evasion mechanisms, upregulate additional immune checkpoints, or proliferate beyond the threshold at which checkpoint blockade can achieve meaningful tumor control (15). This bidirectional theoretical concern provides the biological basis for the clinical intuition that a “window” of optimal timing should exist, even though its precise boundaries remain entirely undefined in prospective data.

### C-Wound healing and the cytokine milieu

Active wound healing engages multiple immunoregulatory pathways that are potentially relevant to ICI activity. Transforming growth factor-beta (TGF-β), a key mediator of wound healing and tissue repair, is known to suppress cytotoxic T-cell function, inhibit T-cell activation, and directly counteract ICI-driven immune activation. Elevated TGF-β signaling in the post-cystectomy wound milieu may therefore attenuate the anti-tumor T-cell response that checkpoint blockade aims to reinvigorate (18). Vascular endothelial growth factor (VEGF), also markedly elevated during wound repair and surgical angiogenesis, contributes to immunosuppressive tumor microenvironments through its inhibitory effects on dendritic cell maturation, T-cell trafficking, and NK cell function (19). Theoretically, initiating ICI therapy against this immunosuppressive cytokine background of early wound healing may attenuate treatment efficacy, though direct clinical evidence for this mechanism specifically in RC patients is currently absent.

It is important to state explicitly that the above biological arguments, while mechanistically coherent, are speculative as they apply to the specific clinical context of adjuvant ICI therapy after cystectomy. No prospective study has directly interrogated how the timing of postoperative ICI initiation interacts with these biological processes in MIBC patients (11). Evidence-free expert inference should not be conflated with established clinical guidance.

## What have clinical trials actually studied?

### A-CheckMate 274

CheckMate 274 was a randomized, double-blind, placebo-controlled phase III trial comparing adjuvant nivolumab (240 mg intravenously every two weeks for 16 cycles) with placebo in patients with high-risk MIBC or UTUC following RC or radical nephroureterectomy. Eligible patients were required to meet one of two pathological criteria: those who had received prior neoadjuvant cisplatin-based chemotherapy were eligible with residual disease of ypT2–ypT4a or ypN+ at cystectomy; those who had not received neoadjuvant cisplatin-based chemotherapy were eligible if they had pT3–pT4a or pN+ disease and were ineligible for or had declined adjuvant cisplatin-based chemotherapy. Patients with pT2 N0 disease were only eligible if they had received prior neoadjuvant cisplatin-based chemotherapy. Critically, the protocol mandated that randomization occur within 120 days of surgery, a window chosen to allow adequate postoperative recovery while reflecting the investigators’ expectations about practical treatment initiation timelines (4).

The trial met its co-primary endpoints, demonstrating a statistically significant improvement in DFS in the overall population (median 20.8 versus 10.8 months; HR 0.70; 98.22% CI 0.55–0.90; p<0.001) and a more pronounced benefit in PD-L1-selected patients (tumour cell PD-L1 expression ≥1%: HR 0.55; 98.72% CI 0.35–0.85; p<0.001) (4). Updated OS analyzes at three-year follow-up showed a consistent directional survival benefit in the nivolumab arm, though OS had not reached statistical significance in the overall population at the time of reporting (20). Non-urothelial recurrence-free survival was also significantly improved with nivolumab (HR 0.72; 95% CI 0.59–0.88) (4).

A fundamental limitation with respect to the timing question is that CheckMate 274 did not include any pre-specified or exploratory analysis examining whether the DFS benefit varied as a function of the interval between cystectomy and treatment initiation within the 120-day window (4, 11). Accordingly, it is not possible to determine from these data whether patients randomized at 30 days fared differently from those randomized at 90 or 110 days. The 120-day cutoff must therefore be interpreted as an operational eligibility criterion, not as evidence that initiation beyond this point is ineffective, nor as proof that earlier initiation within the window confers superior outcomes.

### B-AMBASSADOR (Alliance A031501)

The AMBASSADOR trial (Alliance A031501) was a randomized, open-label, phase III trial that enrolled patients with histologically confirmed MIUC of the bladder, upper tract, or urethra following RC to adjuvant pembrolizumab (200 mg intravenously every three weeks for 18 cycles) or active observation. Patients were eligible if they had ≥pT2 and/or pN+ or positive surgical margins at cystectomy; those who had received prior neoadjuvant platinum-based chemotherapy were eligible with ≥ypT2 and/or ypN+ or positive margins, while patients who had not received neoadjuvant chemotherapy were required to have ≥pT3 and/or pN+ or positive margins and to be cisplatin-ineligible or to have declined cisplatin-based adjuvant chemotherapy. ECOG performance status of 0–2 was required. Notably, AMBASSADOR was the first adjuvant trial to enroll patients with positive surgical margins, who constituted approximately 2.4% of the enrolled cohort. Study treatment was required to commence within 116 days of surgery, a timeframe almost identical to that of CheckMate 274 (3).

The trial met its primary endpoint, with pembrolizumab significantly improving DFS compared with observation (HR 0.69; 95% CI 0.54–0.89; p=0.007). The DFS benefit was observed consistently across pre-specified subgroups, including patients who had or had not received prior neoadjuvant chemotherapy. OS data remain immature and the final OS analysis is awaited (3).

As with CheckMate 274, no published subgroup analysis of AMBASSADOR has stratified outcomes according to the time from surgery to treatment initiation (3, 11). The near-identical timing windows of the two trials therefore reflect convergent trial design assumptions rather than independent validation of an optimal biological treatment window (11).

### C-IMvigor010

IMvigor010 was a randomized, open-label, phase III trial that enrolled patients with ypT2–T4a or ypN+ urothelial carcinoma after neoadjuvant chemotherapy and RC, or pT3/T4a or pN+ patients who had not received prior neoadjuvant chemotherapy, randomizing them to adjuvant atezolizumab (1200 mg intravenously every three weeks for 16 cycles) versus observation. Randomization was required within 14 weeks (~98 days) of surgery. The trial failed its primary DFS endpoint (HR 0.89; 95% CI 0.74–1.08; p=0.24), representing a negative result that stands in direct contrast to CheckMate 274 and AMBASSADOR (21).

The reasons for this discrepant result remain incompletely understood and are likely multifactorial. A higher proportion of post-neoadjuvant chemotherapy patients in IMvigor010, a population that may have lower residual tumor immunogenicity after prior cytotoxic treatment, and differences in the checkpoint pathway targeted (PD-L1 blockade with atezolizumab versus PD-1 blockade with nivolumab or pembrolizumab) may have contributed to the negative outcome (22). Differences in tumor mutational burden and CD8+ T-cell infiltration between the enrolled populations may also be relevant (21). Importantly for the timing question, the IMvigor010 eligibility window was slightly shorter than that of CheckMate 274 (~14 weeks versus ~17 weeks), yet this shorter window did not translate into superior efficacy (11). This observation, while not conclusive, does not support the hypothesis that earlier initiation within the eligibility window confers greater benefit.

The most clinically significant finding from IMvigor010 in the context of this review derives from a pre-specified, prospectively planned ctDNA subgroup analysis published by *Powles et al.* (23). Patients with detectable ctDNA after cystectomy had dramatically inferior DFS in the observation arm (median 5.9 months) compared with ctDNA-negative patients (median 24.8 months), confirming the potent prognostic significance of post-cystectomy MRD status. Among ctDNA-positive patients, atezolizumab showed a directional DFS benefit (HR 0.58; 95% CI 0.30–1.11) that did not reach statistical significance owing to limited sample size, while ctDNA-negative patients showed no evidence of benefit from adjuvant ICI treatment (23). Although this analysis was underpowered, it provides the most compelling proof-of-concept evidence to date that patient stratification by MRD status, rather than uniform calendar-guided treatment, may represent the most biologically rational approach to adjuvant ICI use in MIBC.

### D-NIAGARA and recent peri-cystectomy trials

The NIAGARA trial evaluated a perioperative durvalumab strategy: neoadjuvant durvalumab combined with gemcitabine-cisplatin chemotherapy for four cycles, followed by RC, followed by adjuvant durvalumab monotherapy for eight cycles, compared with chemotherapy alone in cisplatin-eligible patients with cT2–T4a N0/N1 MIBC. The trial demonstrated a statistically significant improvement in event-free survival (EFS) with the perioperative strategy (HR 0.68; 95% CI 0.56–0.82; p<0.001) and an improvement in pathological complete response (pCR) rates with the durvalumab-containing arm (7).

NIAGARA does not directly address the question of adjuvant-only timing, as the study was not designed to isolate the contribution of the adjuvant durvalumab phase from the neoadjuvant component. However, the positive result with a peri-surgical ICI strategy, one that encompasses both neoadjuvant immune priming before surgery and adjuvant consolidation thereafter, provides conceptual support for continuous or near-continuous immune engagement across the surgical boundary (7). This observation, combined with the preclinical data suggesting that neoadjuvant ICI may establish durable tumor-specific T-cell memory, raises the hypothesis that the immunological context at the time of adjuvant ICI initiation may be meaningfully influenced by prior neoadjuvant immune priming, a variable absent from the CheckMate 274 and AMBASSADOR populations (15).

Several additional perioperative trials have further expanded the peri-cystectomy immunotherapy landscape. The phase III VOLGA trial (NCT04960709) evaluated perioperative durvalumab combined with neoadjuvant enfortumab vedotin (EV), with or without the anti-CTLA-4 antibody tremelimumab, versus cystectomy with or without approved adjuvant therapy, in 695 cisplatin-ineligible or cisplatin-declining patients with cT2–T4a N0/N1 MIBC. In a planned interim analysis, the doublet of perioperative durvalumab plus neoadjuvant EV demonstrated statistically significant and clinically meaningful improvements in both EFS and OS versus standard of care, and the triplet arm (with tremelimumab) also improved EFS significantly, with OS data not yet crossing the prespecified boundary at the interim analysis (8). These results introduce a chemotherapy-free perioperative option for cisplatin-ineligible patients and add to the biological rationale for perioperative immune engagement, although the relative contribution of neoadjuvant versus adjuvant treatment phases cannot be isolated from this design.

The phase III KEYNOTE-905/EV-303 trial (NCT03924895) similarly evaluated perioperative EV plus pembrolizumab, administered as neoadjuvant treatment followed by RC and adjuvant EV plus pembrolizumab for six cycles, then pembrolizumab alone for six additional cycles, versus surgery alone in cisplatin-ineligible or cisplatin-declining patients with MIBC. The trial demonstrated statistically significant improvements in EFS (HR 0.40), OS (HR 0.50), and pathological complete response rate, and led to FDA approval of perioperative pembrolizumab plus EV for cisplatin-ineligible MIBC in November 2025 (24). This approval establishes a new perioperative standard for the cisplatin-ineligible population and raises important questions about the optimal timing and duration of the adjuvant immunotherapy component within such combined regimens.

Most recently, the phase III KEYNOTE-B15/EV-304 trial (NCT04700124) extended this perioperative strategy to cisplatin-eligible patients, randomizing them to perioperative EV plus pembrolizumab versus standard neoadjuvant gemcitabine/cisplatin followed by cystectomy. Top-line results from a planned interim analysis, presented at the 2026 ASCO Genitourinary Cancers Symposium, demonstrated statistically significant improvements in EFS, OS, and pathological complete response versus neoadjuvant platinum-based chemotherapy, with regulatory submissions under FDA priority review (10). If confirmed with full regulatory approval, EV-304 would establish a perioperative EV-based regimen as a new standard across both cisplatin-eligible and ineligible populations, fundamentally transforming the peri-cystectomy treatment landscape and making the adjuvant timing question increasingly relevant to integrated perioperative designs rather than adjuvant-only strategies alone.

A summary of the pivotal clinical trials, including their treatment initiation windows, eligibility criteria, and key conclusions regarding adjuvant immunotherapy timing, is provided in **Table 1**.

**Table 1:** Overview of key clinical trials, their timing windows, eligibility criteria, and conclusions relevant to adjuvant immunotherapy timing in MIBC.

Trial	Agent	Timing window after cystectomy	Key eligibility criteria	Timing-specific analyzes (yes/no)	Main conclusions relevant to timing
CheckMate 274	Nivolumab 240 mg Q2W × 16 cycles	Randomization within 120 days (~4 months) of surgery	Post-NAC: ypT2–ypT4a or ypN+ at RC. Without prior NAC: pT3–pT4a or pN+, and cisplatin-ineligible or declined adjuvant cisplatin. High-risk UTUC post-nephroureterectomy included. pT2 N0 eligible only with prior NAC.	No timing-stratified efficacy analysis published	DFS benefit established (HR 0.70 overall; HR 0.55 in PD-L1≥1%) within the 120-day window. Timing window is operationally defined, not biologically validated. ctDNA-positive subgroup derived markedly greater benefit.
AMBASSADOR (Alliance A031501)	Pembrolizumab 200 mg Q3W × 18 cycles	Randomization within 116 days (~4 months) of surgery	Post-NAC: ≥ypT2 and/or ypN+ or positive margins. Without prior NAC: ≥pT3 and/or pN+ or positive margins, cisplatin-ineligible or declined adjuvant cisplatin. ECOG 0–2. First adjuvant trial to enrol positive surgical margins.	No timing-stratified efficacy analysis published	DFS improvement (HR 0.69; p=0.007). Timing window mirrors CheckMate 274 but reflects administrative design. No evidence for a biological threshold. OS analysis pending.
IMvigor010	Atezolizumab 1200 mg Q3W × 16 cycles	Randomization within 14 weeks (~98 days) of surgery	ypT2–T4a or ypN+ post-neoadjuvant chemotherapy; or pT3/T4a or pN+ without prior chemotherapy; ECOG 0–2	No timing-stratified efficacy analysis published; ctDNA subgroup analysis available (Powles et al., Nature 2021)	Negative primary endpoint (DFS HR 0.89; p=0.24). ctDNA-positive patients showed directional DFS benefit (HR 0.58). Highlights importance of MRD-based patient selection over calendar-guided initiation.
NIAGARA	Durvalumab (perioperative: neoadjuvant + adjuvant)	Adjuvant phase initiated after RC; neoadjuvant component precedes surgery	Cisplatin-eligible cT2–T4a N0/N1 MIBC; no prior systemic therapy	No; perioperative design precludes independent analysis of adjuvant timing	EFS improvement with perioperative durvalumab (HR 0.68; p<0.001). Does not isolate adjuvant-only timing effect. Provides conceptual support for peri-surgical ICI exposure.
IMvigor011	Atezolizumab 1680 mg Q4W × 12 cycles (ctDNA-positive only)	Surveillance enrolment within 6–24 weeks of cystectomy; ctDNA-positive patients randomized (not a fixed calendar window for treatment)	(y)pT2–T4a N0M0 or (y)pT0–T4a N+M0 urothelial carcinoma; no radiographic evidence of disease; ECOG 0–2. ctDNA positivity confirmed by serial Signatera testing (tumour-informed assay).	Yes ctDNA-positivity prospectively used to select patients for randomization; ctDNA status is the treatment-allocation biomarker	First positive phase III ctDNA-guided adjuvant ICI trial in MIBC. DFS improved in ctDNA-positive patients (HR 0.64; 95% CI 0.47–0.87; p=0.0047); OS reduced by 41%. ctDNA-negative patients had excellent outcomes without treatment (2-yr DFS 88.4%; 2-yr OS 97.1%). Supports MRD-directed rather than exclusively calendar-directed treatment strategies.
VOLGA	Neoadjuvant durvalumab + EV ± tremelimumab → RC → adjuvant durvalumab ± tremelimumab (perioperative)	Perioperative design; adjuvant timing not independently specified	cT2–T4a N0/N1 MIBC; cisplatin-ineligible or declined; ECOG 0–2; candidates for RC	No; perioperative design precludes independent analysis of adjuvant timing	Perioperative durvalumab + neoadjuvant EV significantly improved EFS and OS vs SOC (cisplatin-ineligible). Triplet (+ tremelimumab) improved EFS; OS data not yet statistically significant at interim. First chemotherapy-free perioperative strategy to demonstrate EFS and OS benefit in cisplatin-ineligible MIBC.
KEYNOTE-905/EV-303	EV + pembrolizumab (neoadjuvant → RC → adjuvant EV + pembrolizumab ×6 cycles, followed by pembrolizumab ×6 cycles)	Perioperative design; adjuvant timing not independently specified	cT2–T4a N0/N1 MIBC (≥50% urothelial histology); cisplatin-ineligible or declined; candidates for RC; ECOG 0–2	No; perioperative design precludes independent analysis of adjuvant timing	Significant improvements in EFS (HR 0.40), OS (HR 0.50), and pCR vs surgery alone. Led to FDA approval (November 2025) of perioperative EV + pembrolizumab for cisplatin-ineligible MIBC. First perioperative ADC-ICI regimen with proven OS benefit in cisplatin-ineligible population.
EV-304/KEYNOTE-B15	EV + pembrolizumab (neoadjuvant → RC → adjuvant EV + pembrolizumab) vs neoadjuvant GC → RC	Perioperative design; adjuvant timing not independently specified	cT2–T4a N0/N1 MIBC (≥50% urothelial histology); cisplatin-eligible (no Galsky ineligibility criteria met); ECOG 0–1; candidates for RC + PLND	No; perioperative design precludes independent analysis of adjuvant timing	Significant improvements in EFS, OS, and pCR vs neoadjuvant GC + surgery in cisplatin-eligible patients. First non-platinum perioperative regimen to surpass cisplatin-based NAC in this population. FDA priority review granted (PDUFA date August 2026). If approved, would establish EV + pembrolizumab as a perioperative option regardless of cisplatin eligibility.

RC, radical cystectomy; MIBC, muscle-invasive bladder cancer; UTUC, upper tract urothelial carcinoma; DFS, disease-free survival; EFS, event-free survival; OS, overall survival; ICI, immune checkpoint inhibitor; ctDNA, circulating tumour DNA; PD-L1, programmed death-ligand 1; HR, hazard ratio; MRD, minimal residual disease; Q2W, every two weeks; Q3W, every three weeks; Q4W, every four weeks; EV, enfortumab vedotin; ADC, antibody–drug conjugate; GC, gemcitabine/cisplatin; NAC, neoadjuvant chemotherapy; PLND, pelvic lymph node dissection; SOC, standard of care; pCR, pathological complete response.

## Lessons from adjuvant chemotherapy timing

In the absence of prospective timing data from ICI trials, oncologists have frequently drawn analogies with the established literature on adjuvant chemotherapy timing after RC. Retrospective and population-level studies have consistently suggested that delaying cisplatin-based adjuvant chemotherapy beyond 90–120 days after cystectomy is associated with inferior survival outcomes (25). Evidence regarding the impact of delayed adjuvant chemotherapy initiation after radical cystectomy is derived primarily from retrospective analyzes. A National Cancer Database study demonstrated that adjuvant chemotherapy was associated with an overall survival benefit even when initiated 91–180 days after surgery, while an international multicenter analysis (RISC) found no significant difference in progression-free or overall survival between patients starting chemotherapy within or beyond 90 days, although initiation within 90 days remains the timeframe adopted in most clinical trials (26, 27). These findings have been broadly interpreted as establishing a pragmatic upper limit for adjuvant therapy initiation after RC.

However, the validity of extrapolating these observations to ICI therapy is limited by fundamental mechanistic differences. Cytotoxic chemotherapy exerts its primary anti-tumour effect through direct tumour cell killing; delays allow residual clonogenic cells to proliferate and accumulate additional resistance mutations, progressively undermining the therapeutic window. Immunotherapy, by contrast, operates through the induction and reinvigoration of anti-tumour T-cell responses, a process fundamentally dependent on T-cell receptor diversity, PD-1/PD-L1 axis expression levels, tumour antigen presentation, and the immunogenicity of residual tumour deposits, all of which evolve dynamically in the postoperative period (22). The immunological perturbations of the early postoperative period may theoretically even argue for a brief delay before ICI initiation, the converse of the chemotherapy paradigm, though this remains entirely speculative in the absence of direct clinical data (16).

In summary, while the adjuvant chemotherapy literature provides a reasonable pragmatic framework against excessive delays in initiating post-cystectomy systemic therapy, it does not provide a validated or mechanistically transferable template for ICI timing decisions, and its findings should not be directly applied to immunotherapy decision-making without explicit acknowledgement of these limitations.

## The emerging role of ctDNA and minimal residual disease

### A-Prognostic significance of postoperative ctDNA

ctDNA, derived from tumor cells and detected through analysis of tumor-specific somatic variants in plasma cell-free DNA, has emerged as a sensitive and specific biomarker of MRD following curative-intent surgery in multiple solid tumor types. In colorectal cancer, ctDNA positivity post-resection has been shown to predict disease recurrence with high sensitivity and to guide adjuvant chemotherapy decision-making in prospective trials (28). In urothelial carcinoma specifically, post-cystectomy ctDNA positivity serves as a surrogate indicator of persistent systemic micrometastatic disease, the precise substrate against which adjuvant therapy is directed (23, 29). Patients who are ctDNA-positive following RC represent a biologically distinct, high-risk cohort with markedly elevated relapse rates compared with ctDNA-negative counterparts, irrespective of pathological stage (23).

### B-ctDNA evidence from CheckMate 274

An exploratory *post hoc* biomarker analysis from the 5-year follow-up of CheckMate 274 demonstrated that baseline circulating tumor DNA (ctDNA) status following radical surgery was a powerful prognostic biomarker. Detectable ctDNA identified patients at substantially higher risk of recurrence, with a median disease-free survival (DFS) of 5.0 months compared with 52.1 months in patients with undetectable ctDNA (HR 0.30; 95% CI 0.18–0.48). Among patients with detectable ctDNA, adjuvant nivolumab was associated with a marked improvement in DFS compared with placebo (HR 0.35; 95% CI 0.18–0.66), whereas no clear benefit was observed in patients with undetectable ctDNA (HR 0.99; 95% CI 0.51–1.93). Although these findings are exploratory and derived from a *post hoc* analysis of a relatively small biomarker-evaluable cohort, they suggest that ctDNA may help identify patients most likely to benefit from adjuvant immunotherapy and support the development of biomarker-guided treatment strategies pending prospective validation (30).

### C-ctDNA evidence from IMvigor010

The ctDNA subgroup analysis from IMvigor010, published by *Powles et al.* in *Nature*, remains the most detailed exploration of MRD-guided adjuvant ICI decision-making in urothelial carcinoma. ctDNA was detectable at study entry in approximately 37% of enrolled patients. ctDNA-positive patients in the observation arm experienced dramatically inferior DFS (median 5.9 versus 24.8 months in ctDNA-negative patients), confirming the strong and independent prognostic significance of post-cystectomy MRD status. The directional DFS benefit of atezolizumab in ctDNA-positive patients (HR 0.58; 95% CI 0.43–0.79), despite the overall trial being negative, suggests that the primary negative result of IMvigor010 may in part reflect dilution of the treatment signal by the inclusion of ctDNA-negative patients unlikely to harbour residual disease. Serial ctDNA measurement further identified patients who spontaneously cleared ctDNA during observation, so-called clearers, who had markedly better outcomes than patients with persistent ctDNA positivity, irrespective of treatment assignment (23, 31). Practical barriers also remain relevant to routine implementation. Access to tumour-informed ctDNA assays is not universal, testing may entail substantial cost and variable reimbursement, and differences between platforms in analytical performance and reporting complicate standardization. In addition, assay turnaround time may limit the ability to use ctDNA results for time-sensitive postoperative treatment decisions (31).

## Biomarker-guided timing: a future paradigm?

Taken together, these data raise the conceptually attractive possibility of a ctDNA-guided adjuvant treatment strategy in which: (1) patients are monitored with serial ctDNA testing after cystectomy; (2) treatment is initiated upon ctDNA positivity rather than at a fixed calendar time point; and (3) ctDNA clearance serves as a dynamic response biomarker (23, 29). This approach would rationalize treatment initiation on biological grounds rather than administrative ones, potentially sparing ctDNA-negative patients from treatment-related toxicity while ensuring that ctDNA-positive patients receive therapy at the earliest detectable signal of residual disease (32).

However, several obstacles have historically limited the implementation of this paradigm, and while IMvigor011 has now demonstrated the proof of concept, further challenges persist. First, until IMvigor011, no prospective randomized trial had validated ctDNA-directed adjuvant therapy in MIBC as superior to the calendar-guided approach; that gap has now been addressed in principle, though generalizability of the Signatera assay platform and the specific atezolizumab regimen remains to be established (27). Second, ctDNA assay platforms vary substantially in sensitivity, specificity, limit of detection, and analytical methodology; no universal standard has been established or endorsed by regulatory authorities (33). Third, the optimal timing, frequency, and specimen type for post-cystectomy ctDNA sampling have not been prospectively defined. Fourth, the clinical implications of spontaneous ctDNA clearance, and whether late clearers derive benefit from delayed ICI initiation, are incompletely understood. These limitations are significant but not insurmountable. The landmark phase III IMvigor011 trial (NCT04660344) has now provided the first prospective randomized validation of a ctDNA-guided adjuvant strategy in MIBC. In IMvigor011, 761 patients with histologically confirmed (y)pT2–T4a N0M0 or (y)pT0–T4a N+M0 urothelial carcinoma and no radiographic evidence of disease were enrolled in surveillance within 6 to 24 weeks of cystectomy and underwent serial Signatera ctDNA testing every six weeks for up to one year. Patients with confirmed ctDNA positivity (n=250) were randomized 2:1 to adjuvant atezolizumab (1680 mg every four weeks for 12 cycles) or placebo; ctDNA-negative patients were followed without treatment. Among ctDNA-positive patients, atezolizumab significantly improved DFS (median 9.9 versus 4.8 months; HR 0.64; 95% CI 0.47–0.87; p=0.0047) and reduced the risk of death by 41% versus placebo. In striking contrast, ctDNA-negative patients had a two-year DFS of 88.4% and a two-year OS of 97.1% without any adjuvant treatment, confirming that MRD-negative patients may be safely observed rather than uniformly treated. IMvigor011 directly emerged from the exploratory ctDNA findings of IMvigor010, representing the first successful translation of biomarker-hypothesis into a positive biomarker-selected phase III trial in MIBC, and marks a conceptual transition from pathology-guided uniform adjuvant treatment to biologically-guided MRD-directed therapy (34). Moreover, ongoing ctDNA-directed trials, including TOMBOLA and MODERN, will provide additional prospective evidence to further define which patients benefit from ctDNA-directed strategies (35, 36).

## Current guideline interpretation

The European Association of Urology (EAU) guidelines on muscle-invasive and metastatic urothelial carcinoma currently recommend offering adjuvant nivolumab to eligible patients with high-risk pathological features following RC, based primarily on the CheckMate 274 data. The EAU guidance endorses initiation within the trial-defined eligibility window and does not extrapolate recommendations beyond the studied population (5). The National Comprehensive Cancer Network (NCCN) guidelines similarly designate adjuvant nivolumab (following RC) and adjuvant pembrolizumab as Category 1 recommendations for patients with pT3/T4 N0 or pN+ MIBC who are cisplatin-ineligible or have declined adjuvant chemotherapy, with guidance reflecting the trial-defined treatment initiation windows (6).

It is of paramount importance to interpret these guideline recommendations accurately. The endorsement of treatment initiation within approximately 120 days reflects the eligibility boundaries of the pivotal trials that generated the evidence on which regulatory approvals were based, it does not constitute a prospectively validated proof that treatment initiated at day 121 or beyond is biologically ineffective, nor does it endorse day 28 as superior to day 84. Clinicians must understand that guideline recommendations in this area are necessarily extrapolated from eligibility criteria and not from timing-specific efficacy analyzes, which, as reviewed above, have not been performed.

This distinction matters clinically. Patients experiencing prolonged postoperative recovery, surgical complications, anastomotic leaks, or extended hospitalization are frequently unable to initiate therapy within the arbitrarily defined 120-day window (12, 13). Whether such patients should nonetheless be offered adjuvant ICI therapy (and what the likely benefit-risk ratio is) cannot be determined from existing randomized data (3, 4). In the absence of evidence that efficacy is lost beyond this window, a pragmatic and individualized approach, informed by clinical judgement, performance status recovery, and shared decision-making with the patient, is warranted.

## Key unanswered questions

The first critical unanswered question concerns whether earlier initiation within the trial-defined window confers superior outcomes compared with later initiation, specifically, whether treatment at 4–8 weeks is more effective than initiation at 8–16 weeks. The biological arguments for both positions have been presented above, but the clinical data to resolve this question are entirely absent (12, 13). Addressing this would require a pre-specified or exploratory subgroup analysis within one of the major adjuvant trials, comparing outcomes by time-to-treatment quartile or interval category, analyzes that have not been published to date.

The second question is whether meaningful efficacy is lost beyond the 120-day window. From a tumour-biological perspective, there is no compelling evidence that the mechanisms underlying ICI efficacy in MRD eradication are strictly time-limited within the range of 3–6 months after RC (14, 15). The dynamic nature of postoperative immune reconstitution and the evolving T-cell repertoire make confident predictions impossible without direct prospective data. The rapid proliferative capacity of micrometastatic urothelial carcinoma and the risk of acquisition of immune-evasive phenotypes during unimpeded growth represent the principal arguments against excessive delay, but the precise timeframe at which this risk becomes clinically significant has not been established (11, 15, 22).

The third question, whether timing should become ctDNA-guided rather than calendar-guided, is arguably the most transformative but also the most distant from current clinical implementation. A ctDNA-guided approach would represent a fundamental paradigm shift, decoupling adjuvant treatment initiation from the surgical calendar and anchoring it instead to biological evidence of residual disease (23, 30). This approach has now received prospective validation in IMvigor011, and ongoing trials including TOMBOLA and MODERN will provide further evidence to define the optimal ctDNA-directed approach across different patient populations (34–36).

The fourth and most far-reaching question is whether MRD-driven strategies will ultimately supersede the current uniform adjuvant approach entirely. If ctDNA-negative patients derive minimal or no benefit from adjuvant ICI therapy, a hypothesis consistent with the IMvigor010, IMvigor011 and CheckMate 274 biomarker subgroup data, the current practice of treating all high-risk patients uniformly based on pathological stage alone may represent both over-treatment of ctDNA-negative patients and a missed opportunity for more targeted risk stratification (23, 30, 34). This would have profound implications not only for the timing of therapy but for patient selection criteria, treatment duration, and health economic considerations.

## Perspective and conclusion

The central conclusion of this review is that no validated optimal timing threshold for adjuvant immunotherapy after radical cystectomy currently exists. The approximately 120-day windows used in CheckMate 274 and AMBASSADOR define the populations in which efficacy has been demonstrated but were not designed to compare different treatment-initiation intervals (3, 4).

Available evidence supports initiating adjuvant ICI therapy within the studied timeframe, broadly, within approximately four months of RC, when clinically feasible (3, 4). This recommendation is appropriate because it reflects the population in which efficacy has been demonstrated and regulatory approval granted. Patients who are unable to commence therapy within this window due to medical or surgical reasons, including prolonged ileus, wound complications, or compromised performance status, represent an unaddressed clinical scenario for which no evidence-based guidance exists; in such cases, clinical judgement, individualized risk-benefit assessment, and shared decision-making with the patient must prevail.

The biological arguments for a timing-dependent therapeutic window are mechanistically plausible. Surgery-induced immune dysregulation and MDSC expansion, the wound-healing cytokine milieu including TGF-β and VEGF, and the evolving MRD landscape are all theoretically relevant to ICI efficacy in the post-cystectomy period (14, 17–19). Yet none of these biological mechanisms have been prospectively studied in the specific context of adjuvant ICI timing after RC, and the analogy with adjuvant chemotherapy timing, while pragmatically useful, is mechanistically imperfect and should not be over-extrapolated (22, 25).

The most promising path forward lies in the integration of ctDNA and MRD biomarkers into adjuvant treatment algorithms (23, 30). The exploratory ctDNA analyzes from IMvigor010 and CheckMate 274, now prospectively validated by the phase III IMvigor011 trial, collectively establish that residual disease status, not the calendar date of surgery, is the most biologically rational basis for adjuvant treatment decisions (23, 29, 34). IMvigor011 provides the framework for a transition from calendar-guided to ctDNA-guided adjuvant immunotherapy, a shift that addresses not only the timing question but the broader patient selection challenge (34).

Ultimately, the timing of adjuvant immunotherapy after RC represents a major unmet need in MIBC clinical research. Future trial designs in the adjuvant setting should incorporate pre-specified timing analyzes as co-primary or secondary endpoints, and biomarker-stratified randomization, with ctDNA as a prospective stratification factor, should be considered standard. Until such data emerge, the oncology community must resist the temptation to elevate trial eligibility criteria to the status of validated biological thresholds, a conflation that, however intuitively appealing, lacks evidentiary support and may lead to suboptimal clinical decision-making.
